# Performance Evaluation of Cross-Linked Polyethylene Insulation of Operating 110 kV Power Cables

**DOI:** 10.3390/polym14112282

**Published:** 2022-06-03

**Authors:** Man Ding, Weifeng He, Jiahe Wang, Jinpeng Wang

**Affiliations:** College of Energy and Electrical Engineering, Hohai University, Nanjing 210098, China; 211606010029@hhu.edu.cn (W.H.); wangjiahe@hhu.edu.cn (J.W.); wjp2447326754@163.com (J.W.)

**Keywords:** cross-linked polyethylene, high voltage power cable, ageing, performance evaluation

## Abstract

The ageing characteristic of XLPE insulation of operating a 110 kV power cable with different service time is studied in this paper. The microscopic morphology of XLPE films from different cables were characterized by using Differential Scanning Calorimetry (DSC), X-ray Diffraction method (XRD), and Fourier Transform Infrared Spectroscopy (FTIR) methods, and the dielectric, mechanical, and electrical properties of XLPE were also measured. The relationship of several typical property parameters with the cable service time were established, and the ageing mechanism of XLPE insulation of the operating cable was also analyzed. It was found that XLPE insulation would endure a recrystallization process in the initial operation stage during which the microscopic morphology would become more perfect with higher crystallinity and denser crystal structure. Then, the thermal oxidation would dominate the ageing process of XLPE with the molecular chains broken and more micromolecular products generated after the cable had operated for more than 10–15 years. The AC breakdown strength decreases with the increase of cable service time, with lower decreasing rate in the initial operation stage and a larger rate after 10–15 years. The Pearson correlation coefficient between the cable service time with the characteristic parameters were calculated, and some of them were found to be effective to be used as indicators for operation state detection of operating power cables.

## 1. Introduction

With the development of urbanization in the past decades, underground cables are becoming more and more commonly used in power transmission systems, and the requirement for extra high voltage cables is also increasing. The average electric field in the cable insulation is getting higher with the increase of voltage degree and it brings more challenges to the cross-linked polyethylene (XLPE) material, which is most commonly used as the main insulation material in high voltage power cables. XLPE has many advantages in long distance, high voltage, and large capacity power transmission systems, but it is limited to moderately stressed cables with average electric fields of 5–7 kV/mm from the long-term service experiences [1]. For now, many power cables have been operating for decades and are approaching the end of the 30-year design life. Electrical utilities are now confronted with the decisions to maintain, repair, or replace the transmission cable system, and we need to determine the ageing condition of the cable system so as to help to make the right decisions. Thus, an in-depth understanding of degradation mechanisms of cable insulation and also the establishment of a dependable cable life model are required. We need to clear up how the cable, especially the cable insulation, ages, including the ageing factors, ageing mechanisms and rate of ageing, and the failure mechanisms under operating conditions, which differs from that under the accelerate life test in laboratories. Furthermore, the improvement of ageing diagnostic tests and the determination of the criteria for ‘repair, refurbish, or replace’ are also required.

The failure of XLPE cables can be owed to the growth of damage starting from weak points under various ageing factors including thermal, electrical, environmental, and mechanical factors, which can cause irreversible changes in the cable insulation. When the ageing factors interact with contaminants, defects, protrusions, or voids that might have been introduced during material processing and cable manufacturing in the insulation material, ageing would take place. It usually leads to localized changes of the insulation material and subsequent degradation in these localized regions and then propagates through the insulation, ultimately leading to partial discharges and electrical treeing. Various experimental techniques such as Differential Scanning Calorimetry (DSC), X-Ray Diffraction (XRD), Fourier Transform Infrared Spectroscopy (FTIR), Broadband dielectric spectroscopy, and AC breakdown strength measurements are expected to provide physical-chemical, microstructural, and electrical characterization of the XLPE insulation [2]. Additionally, we hope that these experimental techniques can provide ageing markers that can be used for the diagnosis and prognosis of ageing causing the degradation of cables, which can take place in service.

The ageing mechanism of XLPE insulation and diagnosis techniques of high voltage (HV) cable have been studied thoroughly in the past decades, and many theories and models addressing the ageing mechanism and diagnostic methods have been proposed. There have been some typical theories of electro-thermal ageing of XLPE, among which three main proponents are Dissado–Montanari–Mazzanti (DMM) theory [3,4,5,6,7], that of Lewis et al. [8], and the Crine theory [9]. The space charge characteristic in XLPE insulation is regarded as the critical factor of the ageing process, which was found to increase the ageing rate and accelerate the process to premature failure due to the distortion of local electric field. The Lewis model takes electrical-induced electromechanical stress as a negligible factor in PE breakdown, as the semi-crystalline nature of PE makes the morphology complex with considerable non-uniformity on a sub-microscopic scale. Moreover, the electromechanical stress is expected to encourage sub-micro void and crack production, which may lead to electrical ageing and breakdown in the polymer, and modern scanning probe methods and Raman spectroscopy were suggested to investigate the influence of electrical fields on the structure and interfacial properties of PE at the sub-micrometric level [10]. The Crine model takes the microcrack formation in moderate field as a precursor to breakdown, and amorphous phase deformation and weak attraction bonding breaking would take place, resulting in accelerated ageing, when the electrical field reaches the critical value. It regards space charge as the consequence of ageing as it suggests that strong charge injection does not occur until the nano-cavity formation [9,11,12,13].

Accelerated life test combined with physical, chemical, and microscopic analysis in laboratory are widely used to evaluate the ageing status of XLPE insulation, and then the results are extrapolated to the real service cables [1,14,15,16,17]. Various diagnosis and prognosis methods have been investigated on the basis of the failure mechanism and degradation characteristics of XLPE insulated power cables. Artificial Neural Networks (ANN) were used to predict the insulation properties of XLPE insulation in [18], and the prediction under thermal aging using neural networks presents an alternative solution to expensive and time-consuming laboratory experiments. A procedure for life estimation of high voltage AC cables in real operating conditions, i.e., subjected to voltage and load cycles, were proposed in [19], which was proved to be feasible and provides results consistent with the experience gained over the years about cable operation. Partial discharge in a retired cable joint under different voltage and current stages was investigated, and it showed that the ratio of the PD characteristics between an interval time can be used for joint insulation condition assessment by selecting an appropriate quantitative value in [20]. Isothermal relaxation current (IRC) method was found to be applicable to the condition assessment for XLPE insulated HVAC cable in [21]. The ratio of dielectric loss factors (tanδ) under 0.1 Hz and 50 Hz was proved to be a characteristic parameter for the insulation assessment of XLPE cables in [22]. A direct current integrated charge (DCIC-Q(t)) method was proposed in [23], which was found to be a good diagnostic approach of the high voltage alternating current (HVAC) and high voltage direct current (HVDC) insulation. Electrical treeing tests were recommended for the evaluation of voltage endurance characteristics of insulation materials in [24]. An electrical lifetime model based on the analysis of inverse power model (IPM) parameters and the failure mechanism of XLPE cable were established in [25]. A simulation technique was proposed in [26], which allows full-sized insulation lifetime predictions to be made using data obtained from thin film samples. In the previous research about the evaluation and lifetime prediction of XLPE insulated power cables, most of the results are extracted from a limited set of samples with large randomness, which might be a far from accurate diagnosis and prognosis for cables.

This paper characterizes the XLPE insulation properties of seventeen HVAC cables with the service time ranging from 0 year to 25 years by mechanical, physical, chemical, and electrical measurements. Thin XLPE films were sliced from the service cable insulation and then tested; the melting and crystallization properties were taken by DSC and XRD measurement; the molecular chains and typical functional groups corresponding to material ageing in XLPE were measured by FTIR method; the dielectric properties and electrical strength of XLPE films were tested by broadband dielectric spectrometer and AC breakdown strength experiment. The aggregation structure, chemical structure, and electrical characteristics of XLPE insulation were derived, and the degradation mechanism of XLPE under operating conditions was extracted for cable insulation under service circumstances and some feasible indicators for the insulation assessment of service-aged XLPE cable.

## 2. Samples and Experimentation

### 2.1. Sample Preparation

Seventeen 110 kV commercial power cables that have not reached the end of life were used in this paper, and the service time of the cables is shown in Table 1. The XLPE insulation of the cable was sliced along the axial direction into film samples with different thicknesses by JQB-11 cross-linked cable slicer, among which the 0.2 mm thick films were used for broadband dielectric spectroscopy test and breakdown experiment, the 0.5 mm thick films were prepared for DSC, XRD, and FTIR measurements, the dumbbell-shaped samples were for mechanical tests, and all the XLPE samples were taken from the middle part of the cable insulation for consistency. Before test, all the samples were cleaned with anhydrous ethanol and put into a vacuum drying oven under 70 °C for 8 h to eliminate the mechanical stress during slicing.

### 2.2. Experimentation

XLPE films were tested instantly after they were sliced and cleaned, and the test methods and experiment conditions are listed below.

Thermal property: the melting properties were characterized by DSC measurement. XLPE samples weighting 0.5 mg were tested by using TA Q2000 equipment in a nitrogen atmosphere with the temperature increasing from 20 °C to 140 °C at the rate of 10 °C/min.

Crystallization structure: the crystallization structure of XLPE films were measured by XRD method. XLPE films measuring 0.5 mm thick in the size of 1 × 1 cm were tested by using Bruker D8 ADVANCE equipment with the scanning angle ranging from 10° to 30° at the scanning rate of 5°/min.

Chemical structure: the molecular chain characteristic and typical functional groups that might have been generated during ageing of XLPE were detected by FTIR. XLPE films measuring 0.5 mm thick in the size of 2 × 2 cm were tested by using Thermo Scientific Nicolet iS5 equipment, with the wave number ranging from 500 to 4000 cm^−1^ under Attenuated Total Reflectance (ATR) mode.

Dielectric property: the dielectric constant and dielectric loss factor of XLPE samples were measured by broadband frequency dielectric spectroscopy. XLPE films measuring 0.2 mm thick in the size of 4.5 × 4.5 cm were measured by using Novocontrol Concept 40 in the frequency range of 10^−1^ to 10^7^ Hz, and circular gold electrodes with a diameter of 3 cm and 4 cm were sputtered respectively on the two sides of the films before measurement.

Electrical strength: the electrical strength of XLPE samples were characterized by AC breakdown measurement. XLPE films measuring 0.2 mm thick in the size of 4 × 4 cm were sandwiched between two cylindrical electrodes with the diameter of 2.5 cm and tested under gradually increasing AC voltage at the rate of 2 kV/s until breakdown occurred.

Mechanical property: the elongation at break and tensile strength characteristics were tested by using an insulating tensile testing machine on dumbbell-shape XLPE film with a thickness of 1.2 mm and length of 20 mm.

## 3. Experimental Results

### 3.1. Thermal Property of XLPE Insulation

**Figure 1:**
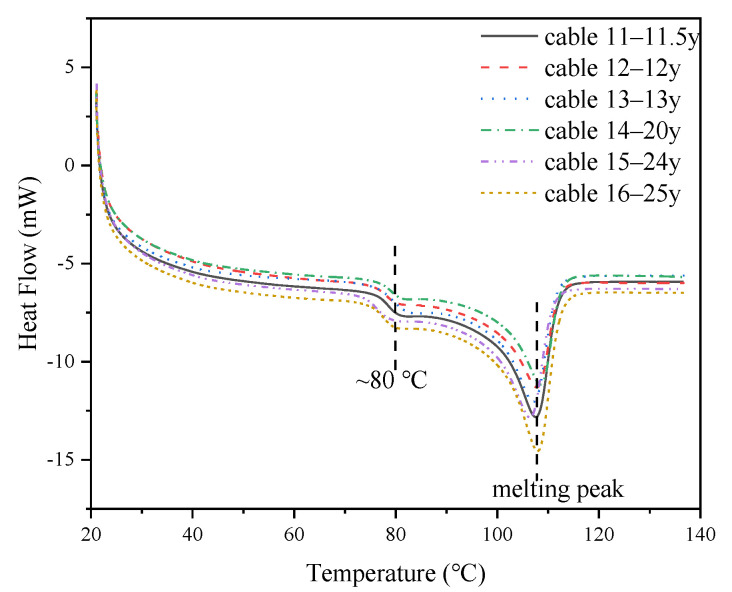
DSC results of XLPE films from cables with different service times.

The melting process of XLPE films were measured, and the results are shown in Figure 1. There are two dominant melting peaks on the DSC spectrum located at around 80 °C and 110 °C. The melting peak at around 80 °C might have been introduced by the degassing process after crosslinking during the manufacturing of XLPE cables, and the 110 °C peak corresponds to melting of the crystals in this half-crystallized polymer.

**Figure 2:**
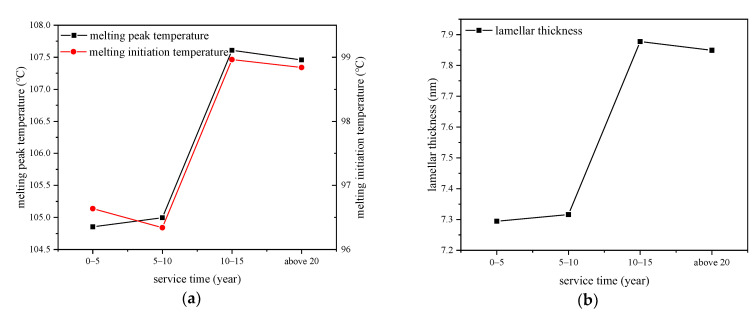
Relationship curves of melting range and crystal lamellar thickness with service time. (**a**) Melting peak and initiation temperatures; (**b**) lamellar thickness.

The melting temperature and initial melting temperature are found out from the DSC spectrum and the relationship with service time are shown in Figure 2. The service time is divided into several year periods to study the influence of operation time to the thermal property of XLPE insulation. The crystal lamellar thicknesses of XLPE samples are calculated by using Equation (1), and the result is also shown in Figure 2.

(1) $$L=\frac{2\sigma_{e}\cdot T_{m0}}{\Delta H_{m0}\cdot (T_{m0}-T_{m})}$$

where *σ_e_* is the surface energy per unit area of the base plane, which is 9.3 × 10^−2^ J·m^−2^, *T_m_* is the melting peak temperature from DSC spectrum of the XLPE samples, *T_m_*_0_ is equilibrium melting temperature of infinite thick crystal, which is 414.6 K; Δ*H_m_*_0_ is the melting enthalpy per unit volume of XLPE crystal that is 2.88 × 10^8^ J·m^−3^; *L* is the lamella thickness.

We can see from Figure 2 that the melting peak temperature and initial melting temperature first increase and then decrease with the increase of service time, and the maximum value is detected from cables that have operated for 10–15 years. The standard deviations of the melting peak temperature, melting initiation temperature, and lamellar thickness of 10–15-year cables are 0.096, 0.14, and 0.022, which are very low compared with the average values. The lamellar thickness has a similar trend with the melting temperature which first increases then decreases with the increase of cable service time, implying that thick lamellar were generated during cable operation, which can be attributed to the ordered arrangement of macromolecular chains in a certain service period of cables, and then the macromolecular chains would have been broken under thermal oxidation which would destroy the original ordered arrangement. That is to say, the crystal structure of XLPE had been optimized during a certain period of cable operation and then might be broken after that, and deformation of polymer processes increases with respect to cable service time.

### 3.2. The Crystalline Structure of XLPE Samples

**Figure 3:**
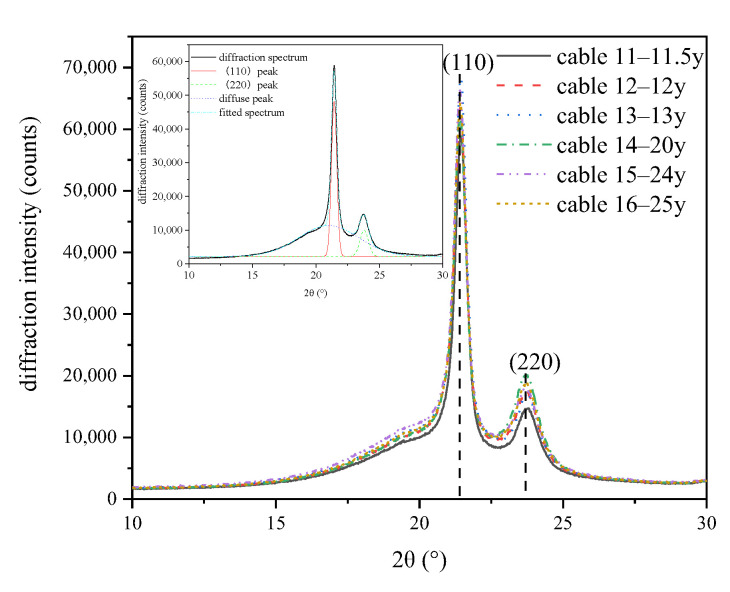
The XRD spectrum of XLPE samples of cables with different service time.

The crystalline structure was tested by using the XRD method, and the diffraction spectrums of XLPE samples from cables with different service times are shown in Figure 3. We can see two sharp peaks located at around 2θ = 21° and 2θ = 23° as well as one diffuse peak located at around 2θ = 20°, indicating the coexistence of crystalline state and amorphous state inside XLPE. The two diffraction peaks at ~21° and ~23° match the (110) and (220) crystal planes, respectively.

The interplanar spacing value between two crystal planes as well as the crystallinity of the XLPE can be calculated by using Equations (2) and (3).

(2) 2dsinθ=nλ

where *d* is the crystalline interplanar spacing between two crystal planes, *θ* is the angle of the diffraction peak and the unit is °, *λ* is the wavelength of X-ray, which is 0.15406 nm, and *n* is the diffraction order which is 1.

(3) $$X=\frac{area2+area3}{area1+area2+area3}\times 100\%$$

where *X* is the crystallinity of XLPE films, *area*1, *area*2, and *area*3 are the areas of the diffraction peaks relating to the amorphous state, (110) and (220) crystal planes, respectively.

**Figure 4:**
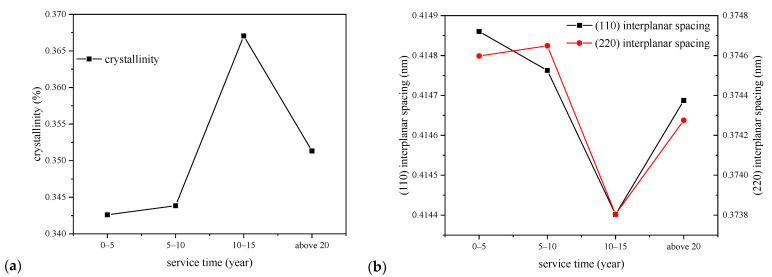
The characteristic parameters of the crystal structure of XLPE films with the service years. (**a**) crystallinity; (**b**) interplanar spacing of each crystal face.

The calculated crystallinity and interplanar spacing values of XLPE samples with the relationship of service years are shown in Figure 4.

From Figure 4, we can see that the crystallinity of XLPE films first increases and then decreases with the increase of cable service year, and the XLPE samples from cables that had operated for 10–15 years shows the maximum crystallinity. The standard deviations of the crystallinity, (110) and (220) interplanar spacings of 10–15-year cables are 1.37%, 0.0002, and 0.00024, which are very low compared with the average values. The interplanar spacings of (110) and (220) crystal planes show similar trends with the crystallinity, which first decrease and then increase with the increase of service year, and also the XLPE samples from cables that had operated for 10–15 years give the minimum interplanar spacing value. The crystallinity increases and interplanar spacing decrease demonstrate that the crystallization structure might have been optimized in the initial operating stage corresponding to the recrystallization process, which was found in accelerated life tests. The XLPE insulation in operating cables endures a continuous low thermal stress, which can facilitate the crystallization process of the material in the initial operating stage. However, the crystallization structure would then be damaged resulting from the thermal oxidation of the XLPE insulation when cables operate longer, which might be the reason for the crystallinity decrease and interplanar spacing increase of XLPE samples from cables that have operated for longer than 15 years in Figure 4.

### 3.3. The Molecular Chain Structure of XLPE Films

**Figure 5:**
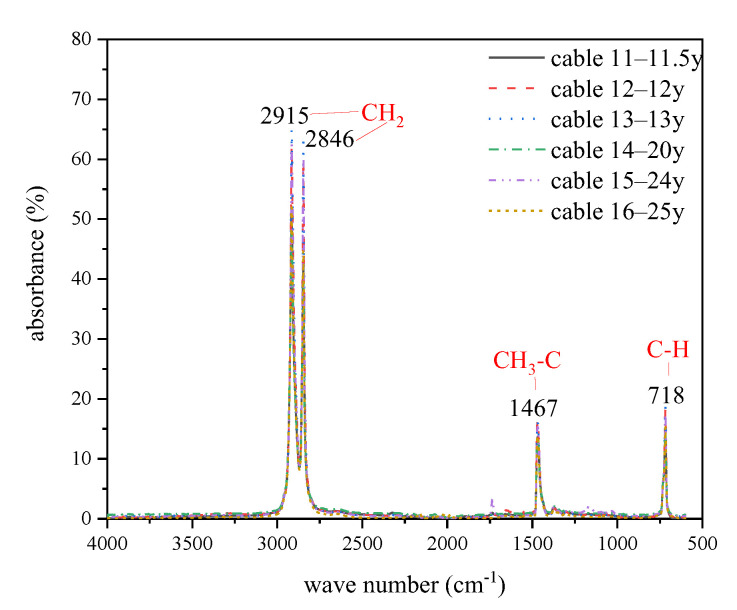
FTIR spectrums of XLPE samples.

The FTIR spectrums of XLPE samples are shown in Figure 5, and there are several obvious absorption peaks located at around 718 cm^−1^ and 1467 cm^−1^ for -CH_2_ bending vibration absorption peaks, 2846 cm^−1^ and 2915 cm^−1^ for -CH_2_ stretching vibration absorption peaks.

**Figure 6:**
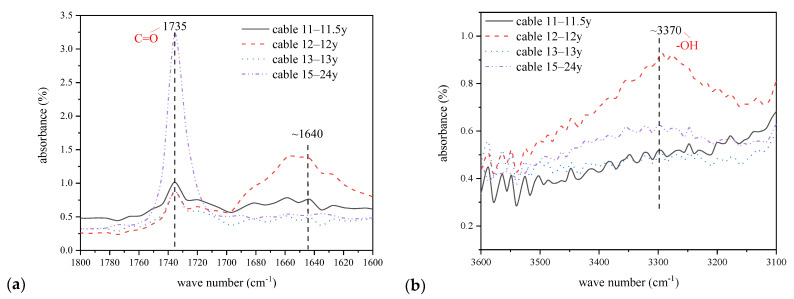
Detailed FTIR spectrum. (**a**) 1600–1800 cm^−1^; (**b**) 3100–3600 cm^−1^.

The detailed spectrum ranging from 1600–1800 cm^−1^ and 3100–3600 cm^−1^ are shown in Figure 6, illustrating the absorption peaks at ~1640 cm^−1^, ~1690 cm^−1^, ~1740 cm^−1^, and ~3370 cm^−1^ corresponding to the styrene, acetophenone, carbonyl, and cumyl alcohol, respectively. Part of these functional groups were generated during the decomposition of dicumyl peroxide (DCP) in the manufacturing of power cables, which would be released gradually during the operation of cables under high voltage, such as acetophenone and cumyl alcohol. On the other hand, the carbonyl and styrene would be generated after a certain period of cable operation owing to the broken of macromolecules by thermal oxidation of XLPE insulation [24]. Thus, the change of the functional group contents that were figured out from the FTIR spectrum can reflect the ageing status of XLPE insulation of power cables.

**Figure 7:**
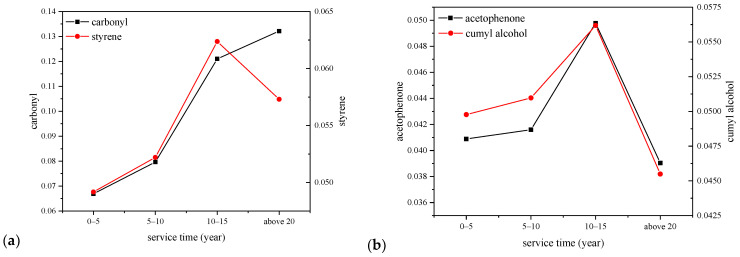
Typical functional group characteristics with the relationship of cable service year. (**a**) Carbonyl and styrene; (**b**) Acetophenone and cumyl alcohol.

We take the peak intensity of methylene at ~718 cm^−1^ as a reference, which hardly changes, and the specific value between the peak intensity of each functional group and the reference peak were calculated and shown in Figure 7.

We can see from Figure 7 that the functional group contents of acetophenone and cumyl alcohol both decrease after a long operation term, and the carbonyl content increases with the increase of cable service years. The standard deviation of the carbonyl index of 10–15-year cables is 0.01, which is very low compared with the average value. This can be attributed to the release of cross-linking by-products during cable operation under high voltage and a certain degree of thermal stress. The increase of carbonyl content is probably resulted from the thermal oxidation of XLPE insulation with macromolecular chains broken to micromolecular products featured by the carbonyl groups. The thermal oxidation process and relevant micromolecular group generation would be justified by the dielectric properties of XLPE films hereinafter.

### 3.4. Dielectric Properties of XLPE Films

**Figure 8:**
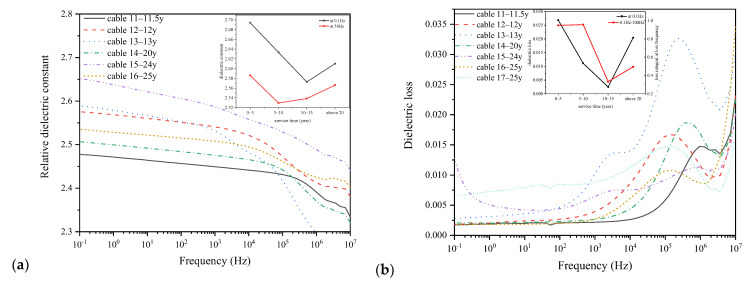
Dielectric properties of XLPE films of cables with different service years. (**a**) Relative dielectric constant; (**b**) dielectric loss.

The frequency domain dielectric spectroscopies of XLPE films of cables with different service years are shown in Figure 8, which were taken at the frequency ranging from 10^−1^ to 10^7^ Hz. Figure 8a shows the relative dielectric constant, and Figure 8b shows the dielectric loss of XLPE samples. The insets of Figure 8 give the relationship between characteristic parameters of the dielectric property and the cable service time.

From Figure 8, we can see that the relative dielectric constant of XLPE film is around 2.3, which decreases gradually with the increase of applied voltage frequency. The relative dielectric constant is determined by the polarization strength of XLPE, which involves in both displacement and relaxation polarization at low frequencies and only displacement polarization at high frequencies, as the orientation of dipoles can hardly keep up with the polarity change of applied voltage at higher frequencies. Thus, the relative dielectric constant decreases with the increase of voltage frequency as shown in Figure 8. On the other hand, the dielectric loss peaks of XLPE films are located at around 10^5^–10^6^ Hz. The inset of Figure 8a shows the relative dielectric constants at low frequency (0.1 Hz) and power frequency (50 Hz) of XLPE films, which first decrease and then increase with the increase of cable service years. Additionally, the trend of low frequency loss and the loss integration in the range of 0.1 Hz to 100 Hz shown in the inset of Figure 8b are similar with the low frequency dielectric constant. The standard deviations of low frequency permittivity and dielectric loss and low frequency dielectric loss integral of 10–15-year cables are 0.045, 1.83 × 10^4^, and 1.62 × 10^3^, which are very low compared with the average values. From the FTIR results of XLPE films in Section 3.3, it was found that there were micromolecular products owing to the macromolecular chain broken during thermal oxidation. The micromolecular products are usually polar groups and could increase the dielectric constant owing to the relaxation polarization.

### 3.5. Mechanical Properties of XLPE Films

**Figure 9:**
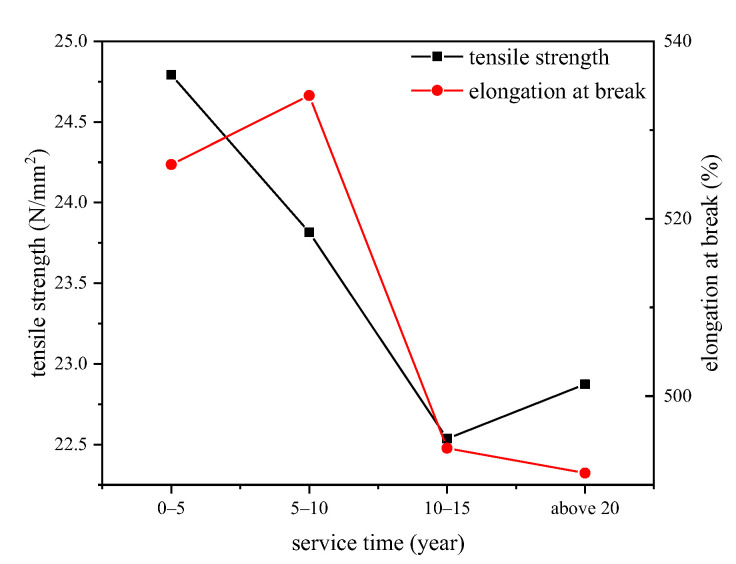
Minimum tensile strength and elongation at break value of XLPE films.

The mechanical properties of XLPE films were tested, and the minimum tensile strength and elongation at break with the relationship of cable services year are shown in Figure 9.

From Figure 9, we can see that the minimum tensile strength value of XLPE films decreases during the operation of cables. The cross-linking by-products such as carbonyl groups or cumyl alcohol groups chaotically distribute inside the amorphous area of XLPE, which hinders the orientation of macromolecular chains along the stretch direction and reduces the tensile strength and elongation at break. On the other hand, the recrystallization procedure of XLPE during which the amorphous area converts to crystal state would increase the mechanical properties. Thus, the change of mechanical properties of XLPE films is the synthetic result of various ageing characteristics.

### 3.6. Electrical Strength of XLPE Films

The breakdown characteristic of dielectrics determines the ability of the dielectric to maintain the insulation performance under electric field, which can be used to predict the service life of the insulating material. The breakdown voltage of insulating material is found to obey Weibull distribution law, so we can use a two-parameter Weibull distribution function to characterize the AC electrical strength of the XLPE films. The breakdown probability of XLPE films under voltage U can be calculated by using Equation (5) according to Weibull distribution function.

(4) $$F(U,\alpha ,\beta )=1-\exp[-{\left(\frac{U}{\alpha }\right)}^{\beta }]$$

where *F* is the breakdown probability of XLPE films under voltage *U*, and *α* and *β* are scale and shape parameters of Weibull distribution.

**Figure 10:**
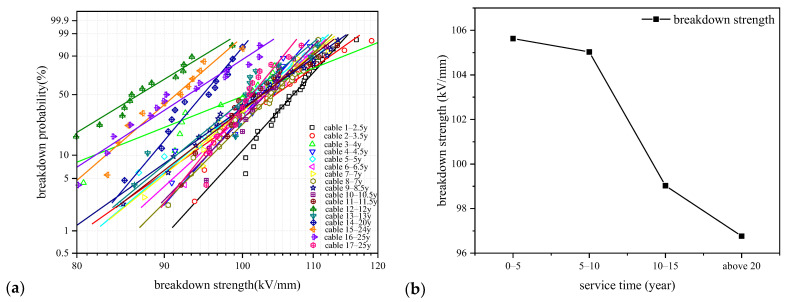
AC breakdown strength of XLPE films. (**a**) Weibull distribution of the breakdown strength of XLPE films of each cable; (**b**) breakdown strengths of XLPE films of each cable.

As the value of Weibull distribution failure probability has great impact on the data processing result, we introduce the Ross distribution function to improve the accuracy of the analysis as shown in Equation (6).

(5) $$F(i,n)=\frac{i-0.44}{n+0.25}$$

where *i* is the serial number of the samples and *n* is the total number of the samples. Arrange the experimental data from small to large and number them accordingly, and Equation (6) gives the failure probability of the sample under the corresponding serial number. We use Ross distribution function to derive the failure probability in this paper, and the breakdown strength when F(t) = 0.632 is used as the breakdown field strength at 63.2% breakdown probability. The AC breakdown strengths of XLPE films are shown in Figure 10.

From Figure 10, we can see that the AC breakdown strength of XLPE films changes little when the service time of cables is no longer than 5–10 years, and it decreases obviously after 10 years of operation. The breakdown strength of XLPE insulation is influenced by the microscopic morphology, including crystallization structure, molecular chain arrangement and defect characteristics in XLPE insulation. From the physical and chemical measurement results in the above, we have studied that the aggregate structure and the molecular chains of XLPE films changes with the cable service time. Recrystallization of amorphous state and release of cross-linking by-products dominate the initial operation stage of cables, and the optimization of the crystal structure and reduction of small molecular polar groups may help to keep the electrical strength of XLPE films. When the cables have operated for more than 10 years, the microscopic morphology would have begun to be damaged and the macromolecular chains would start to break, resulting in the deterioration of XLPE and correspondingly the decrease of electrical strength.

## 4. Discussion

### 4.1. Damage Mechanism of XLPE Insulation during Different Operation Stage

From the performance characterizing results of XLPE samples of cables with different service time, we can see that the physical, chemical, mechanical, and electrical properties of XLPE all change by certain rules under multiple stresses during cable operation.

The aggregate structure shows an optimizing trend before degradation, with the increase of melting temperature, lamellar thickness, and crystallinity and decrease of interplanar spacing.

As we all know, XLPE is a kind of semi-crystalline polymer with crystal area and amorphous area co-existing in it. The crystal area is composed of spherulites with the diameter of dozens of microns generally. Spherulites are formed by stacks of lamellar growing radially from the center nucleus, and the spherulites are spherical in any cases since the lamellar grow at the same rate. In the crystallization process, the molecular chain of XLPE is folded regularly and repeatedly with almost no change in the chain length and bond angle in order to reduce the surface energy to form the lamellar, and the lamellar is about 10 nm thick. The area outside the spherulites and between lamellar is amorphous area, and the percentage of the crystal area is the crystallinity of XLPE. The arrangement of molecular chains is more regular and denser in the crystalline region especially on the lamellar, while it is more chaotic and loose in the amorphous region. Thus, there are more free volumes in the amorphous area, which are cavities without chains (inherent static voids between molecular chains) or a small amount of unoccupied volume at the end of the molecular chain segments [27,28].

Cables operated at temperatures around 60–70 °C, which would promote the XLPE to recrystallize, during which imperfect crystals melt and recrystallize so the crystal structure tends to be more perfect and part of the amorphous area converts to crystallize area, then the crystallinity tends to be higher. The perfection of crystal structure is characterized by larger lamellar thickness, smaller interplanar spacing, and higher crystallinity shown in cables operated for less than 10–15 years in Figure 2 and Figure 4.

After the recrystallization domain process, the ageing of XLPE would be dominated by thermal oxidation process when macromolecular chains are broken and micromolecular products are generated, such as carbonyl and styrene. The amorphous area is firstly damaged because the structure is less dense than that in crystal area, then the lamellar in crystal area subjects to the thermal expansion forces from the amorphous region and the space between lamellar are enlarged. When oxygen enters the spherulites along the interface between amorphous and crystal areas, there would be an oxidation process and then the connection bonding between lamellar and molecular chains inside the lamellar would be broken. Then, the crystal structure is damaged, which is characterized by the decrease of crystallinity and lamellar thickness and increase of interplanar spacing, as shown in cables after running for 10–15 years in Figure 2 and Figure 4 [29].

The dielectric property including relative dielectric constant and dielectric loss shows a similar time-changing law with the aggregation structure, with the dielectric constant and dielectric loss first decreasing and then increasing with the increase of cable service time. In the stage of recrystallization of cables operated for no longer than 15 years, the microstructure of XLPE is optimized and becomes more regular and denser. The small molecular free groups generated during ageing would act as a crystal nucleus and adsorb polymer chains in heterogeneous nucleation to make them orderly arranged and crystallized, thus leading to further crosslinking of polymer chains, improving crystal morphology and promoting post-crosslinking of XLPE [30]. Thus, the dielectric constant and dielectric loss are reduced during the post-crosslinking stage. However, after long term service, XLPE would be thermally oxidized and degrade irreversibly with the increase of dielectric constant and dielectric loss.

The AC breakdown strength of XLPE changes very little in the initial stage of cable operation and then gradually reduces with the increase of cable service time as shown in Figure 10b. The breakdown characteristic would be influenced by the microstructure of XLPE especially by the crystallinity, as the charged particles tend to transport along the amorphous area or the interface between amorphous and crystallized area, implying that larger crystallinity results in a smaller amorphous area, which would block the charge transport and then enhance the breakdown strength correspondingly. On the other hand, small molecular products such as carbonyl and styrene, which are generated during ageing, would increase the number of shallow traps in the amorphous area, which would assist the charge transport and reduce the breakdown strength. Deep traps dominated by the chemical defects that mainly exist in the crystallized area would also act as the trapping centers and facilitate the formation of space charge, and the space charge would distort the local electrical field and then change the breakdown strength of XLPE samples [10].

To summarize, the ageing of XLPE insulation of operating high voltage cables involves in two main physical processes including recrystallization and thermal oxidation in different operation stage. In the recrystallization stage, microstructure of XLPE is optimized with crystallinity increasing and crystal structure more perfect, while in the thermal oxidation stage, the macromolecular chains are broken into micromolecular products and properties would be changed correspondingly along with the degradation of XLPE.

### 4.2. Correlation Analysis of Characteristic Parameters of XLPE Samples

According to the experimental results shown in Section 2, the properties of XLPE change in many ways with the cable service time, and there are several typical performance parameters that can reflect the ageing status of XLPE insulation. The correlation between the cable service time and certain characteristic property parameters are analyzed here.

Correlation analysis is a statistical method to examine the lineal relationship between two variables, and the correlation coefficient determines how one variable changes when related other variables change [31]. The Pearson correlation coefficient can be figured out by using Equation (6).

(6) $$r=\frac{S_{xy}^{2}}{S_{x}S_{y}}=\frac{\sum (x-\bar{x})(y-\bar{y})/n}{\sqrt{\sum {\left(x-\bar{x}\right)}^{2}/n}\cdot \sqrt{\sum {\left(y-\bar{y}\right)}^{2}/n}}=\frac{n\sum xy-\sum x\sum y}{\sqrt{n\sum x^{2}-{\left(\sum x\right)}^{2}}\sqrt{n\sum y^{2}-{\left(\sum y\right)}^{2}}}$$

where *r* is the Pearson correlation coefficient, *x* and *y* are related variables, and *n* is the amount of data of each variable.

The correlation coefficient is between −1 and 1, and the larger the absolute value, the stronger the correlation. The positive and negative of the coefficient represents the correlation direction. The correlation degree in this paper is classified into four degrees: |r| ≤ 0.2 represents very weak correlation or no correlation, 0.2 < |r| ≤ 0.4 represents weak correlation, 0.4 < |r| ≤ 0.6 represents moderate correlation, 0.6 < |r| ≤ 0.8 represents strong correlation, and |r| > 0.8 represents significant strong correlation. When r equals to 1 or −1, it means perfect positive or negative correlation between two variables, respectively.

On the other hand, the P value here is the calculation result of a paired t-test, which is used to estimate the significance degree of the correlation coefficient. The correlation coefficient is proved to be significant when the P value in the Pearson correlation between two parameters is less than 0.05, implying that there is a prominent relationship between the two parameters.

**Figure 11:**
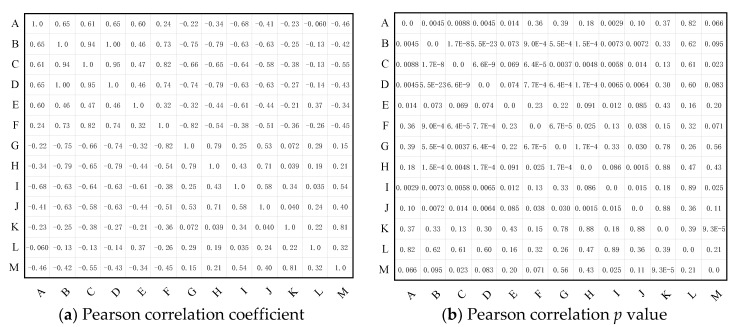
The Pearson correlation analysis between XLPE characteristic parameters. A—service time (year), B—melting temperature (°C), C—initial melting temperature (°C), D—lamellar thickness (nm), E—carbonyl index, F—crystallinity (%), G, H—crystallize interplanar spacing of (110) (220), I—electrical breakdown strength (kV/mm), J—elongation at break (%), K—low frequency dielectric constant, L—low frequency dielectric loss, M—low frequency loss integral.

In this paper, several parameters characterizing the physical, chemical, and electrical properties, including melting temperature, initial melting temperature, lamellar thickness, carbonyl index, crystallinity, crystallized interplanar spacing, electrical breakdown strength, elongation at break, dielectric constant and dielectric loss at 0.1 Hz, and the integral of dielectric loss from 0.1 Hz to 100 Hz, are selected, and the Pearson correlation coefficients between these parameters and cable service time are calculated, which is shown in Figure 11. The *p* value in Figure 11b shows the significance level of the correlations.

We can see that, among all the characteristic parameters, melting temperature, initial melting temperature, lamellar thickness, and carbonyl index are strongly positively correlated with the cable service time, and the electrical breakdown strength is strongly negatively correlated with the cable service time. The elongation at break and dielectric loss integral from 0.1 Hz to 100 Hz are moderately negatively correlated with cable service time. The crystallinity, interplanar spacing of (110) and (220) crystal plane, and dielectric constant at 0.1 Hz are weakly correlated with the cables service time. the dielectric loss at 0.1 Hz is uncorrelated with the cable service time. After the significance test shown in Figure 11b, the relationship between the cable service time and melting temperature, initial melting temperature, lamellar thickness, carbonyl index, and AC breakdown strength are prominent, implying that these characteristic parameters can be considered to be used in the ageing state detection of operating cables.

On the other hand, it was found out that the XLPE insulation would have endured recrystallization during the initial operation stage of cables, so that several ageing parameters change non-monotonically with the increase of cable service time, and the turning point is usually found in cables that had operated for 10–15 years. In other words, the full-scale degradation of XLPE insulation begins after operation about 10–15 years. Thus, the correlation coefficient of cable service year with some of the characteristic parameters would be much larger in cables that have operated for more than 10–15 years, which is figured out to be −0.74 with crystallinity, 0.86 with (110) interplanar spacing and 0.83 with (220) interplanar spacing. That is to say, when cables have been in service over 10–15 years, the aggregation structure of the XLPE would be broken under thermal stress during cable operation, with the obvious decrease of crystallinity and increase of spacing between crystal planes.

To conclude, from the characterizing results of XLPE from high voltage cables with different service time, some characterized parameters including melting temperature, lamellar thickness, carbonyl index and AC breakdown strength can be considered to be used as indicators to detect the ageing status of high voltage cables.

## 5. Conclusions

The ageing characteristics and ageing mechanism of XLPE of operating 110 kV power cables with different service times were studied in this paper; we can conclude it as following:

(1) The microscopic morphology of XLPE insulation was first optimized and then degraded with the increase of cable service time, corresponding to the recrystallization and thermal oxidation process, respectively. The recrystallization process taking place at the initial operating stage of cables is accompanied by the perfection of microstructure with higher crystallinity and denser crystal structure. The dielectric performance is improved with the decrease of low frequency dielectric constant and dielectric loss in the recrystallization process. The AC breakdown voltage decreases with the increase of service time but with much lower decreasing rate during the recrystallization process. After operating for more than 10–15 years, the ageing of XLPE insulation of cables is dominated by thermal oxidation, resulting from the breaking of macromolecular chains and generation of micromolecular products, which would deteriorate the crystal structure and also give rise to the charge traps in amorphous area in XLPE, the dielectric performance and AC breakdown strength both deteriorate obviously then.
(2) Several typical characteristic parameters which can indicate the ageing status of XLPE of operating power cables were selected and the relationship between each parameter and the cable service time were analyzed. The melting temperature, lamellar thickness, carbonyl index, and AC breakdown strength were found to be significantly correlated with the cable service time, which can be considered to be used in the status detection of operating power cables.

The XLPE samples tested in this paper are film samples sliced from the main insulation of power cables, and the characterizing methods maybe something different from that taken directly on cable segments, but the characteristic parameters here selected are good references for the status assessment of high voltage power cables.

## Figures and Tables

**Table 1:** Cable information.

Cable Number	Service Time (Year)	Cable Number	Service Time (Year)
Cable 1	2.5	Cable 10	10.5
Cable 2	3.5	Cable 11	11.5
Cable 3	4	Cable 12	12
Cable 4	4.5	Cable 13	13
Cable 5	5	Cable 14	20
Cable 6	6.5	Cable 15	24
Cable 7	7	Cable 16	25
Cable 8	7	Cable 17	25
Cable 9	8.5		

## Data Availability

The data presented in this study are available on request from the corresponding author. The data are not publicly available due to privacy reasons.
